# The Warburg effect alters amino acid homeostasis in human retinal endothelial cells: implication for proliferative diabetic retinopathy

**DOI:** 10.1038/s41598-023-43022-z

**Published:** 2023-09-25

**Authors:** Andrew Gregory, Thangal Yumnamcha, Mohamed Shawky, Shaimaa Eltanani, Armaan Naghdi, Bing X. Ross, Xihui Lin, Ahmed S. Ibrahim

**Affiliations:** 1 Department of Ophthalmology, Visual, and Anatomical Sciences, School of Medicine, Wayne State University, 540 East Canfield, Gordon Scott Hall (room 7133), Detroit, MI 48201 USA; 2 Department of Biochemistry, Faculty of Pharmacy, Horus University, Damietta, Egypt; 3 Department of Biochemistry, Faculty of Pharmacy, Mansoura University, Mansoura, Egypt; 4 Department of Pharmacology, School of Medicine, Wayne State University, 540 East Canfield, Gordon Scott Hall (room 7133), Detroit, MI 48201 USA

**Keywords:** Biochemistry, Neuroscience, Endocrinology

## Abstract

Proliferative diabetic retinopathy (PDR) remains a leading cause of blindness despite progress in screening and treatment. Recently, the Warburg effect, a metabolic alteration affecting amino acid (AA) metabolism in proliferating cells, has drawn attention regarding its role in PDR. This study aimed to investigate the impact of the Warburg effect on AA metabolism in human retinal endothelial cells (HRECs) subjected to PDR-associated risk factors and validate the findings in patients with PDR. In vitro experiments exposed HRECs to high glucose (HG) and/or hypoxia (Hyp), known inducers of the Warburg effect. The HG + Hyp group of HRECs exhibited significant differences in non-essential AAs with aliphatic non-polar side chains, mainly driven by elevated glycine concentrations. Pathway enrichment analysis revealed several glycine metabolism-related pathways significantly altered due to the Warburg effect induced by HG + Hyp. Crucially, vitreous humor samples from PDR patients displayed higher glycine levels compared to non-diabetic and diabetic patients without PDR. The odds ratio for PDR patients with glycine levels above the cut-off of 0.0836 µM was 28 (*p* = 0.03) compared to non-PDR controls. In conclusion, this study provides mechanistic insights into how a specific Warburg effect subtype contributes to glycine accumulation in PDR and supports glycine's potential as a biomarker for PDR pathogenesis.

## Introduction

The prevalence of diabetes is on the rise globally, and it is projected that more than 50 million Americans will be affected by diabetes by 2030^1^. According to the International Diabetes Foundation Diabetes Atlas, approximately 27% of individuals with diabetes develop diabetic retinopathy (DR), a leading cause of blindness when it progresses to the stage of proliferative DR (PDR)^2^. At the PDR stage, abnormal new blood vessels develop in the retina in a process known as retinal neovascularization (RNV). As time progresses, these abnormal blood vessels can cause hemorrhaging and tractional retinal detachment, ultimately resulting in vision loss^3,4^. The current therapeutic approaches for PDR mainly involve laser photocoagulation and intravitreal injection of anti-vascular endothelial growth factor (VEGF) drugs. However, these therapeutic modalities do not always effectively control the progression of PDR^5^. Moreover, studies have indicated that initiating intravitreal anti-VEGF medication increases the risk of acute thromboembolic events, particularly ischemic strokes^6^. These limitations underscore the need for a comprehensive understanding of the underlying mechanisms of the disease and the identification of new pathways associated with RNV. Such knowledge is crucial for developing alternative or complementary treatment options to effectively address PDR.

A longstanding question in the field of PDR is "Why does RNV develop only after patients have had diabetes for many years?" One possible explanation is that PDR is not a single disease but rather a complex set of disease processes, where hyperglycemia alone is not the sole contributing factor. Hypoxia (Hyp), resulting from tissue ischemia, also plays a significant role and is a common risk factor associated with PDR^7^. Unlike other types of diabetic complications, no rodent model exists to recapitulate all of the stages seen in human PDR. For instance, the existing mouse oxygen-induced retinopathy (OIR) model, induced by oxygen toxicity and relative hypoxia, is utilized to study RNV. However, this model lacks hyperglycemia, which limits its translatability to PDR^8^. Another example is the Akimba mouse model, generated by a cross between the Ins2Akita, the Akita diabetes model, and the trVEGF029 (Kimba) mouse in which VEGF is overexpressed in photoreceptors. The mice in this model manifest hyperglycemia and retinal neovascularization at an earlier age than their parent strains. Additionally, Akimba mice exhibit expedited retinal thinning and photoreceptor loss^9^. Nonetheless, the Akimba model does not account for non-VEGF-related factors that drive angiogenesis in PDR, including hypoxia-driven angiogenic factors such as angiopoietin and erythropoietin^10,11^. Therefore, there is a need to develop a model that combines both hyperglycemia and Hyp to provide a more reliable platform for studying the mechanisms underlying PDR and identifying new pathways that could serve as druggable targets.

Hyp and high glucose (HG) are known stressors that can trigger the Warburg effect^12,13^, which has been recently observed in angiogenic endothelial cells (ECs)^14^. The Warburg effect, originally identified by Nobel Laureate Otto Warburg, involves a metabolic shift characterized by increased glucose uptake and fermentation of glucose into lactate by hyperglycolysis, even under normal oxygen conditions. This metabolic alteration generates an abundance of intermediate metabolites that can be directed towards various pathways to meet the high demands of proliferating cells^15^. One aspect closely related to the Warburg effect is amino acid (AA) homeostasis since AAs are essential for protein synthesis, and angiogenic ECs require an ample supply of AAs to support their rapid growth and proliferation. Each AA has two functional groups (amino and carboxylic acid groups) and a distinctive side chain (R-group) bonded to the alpha-carbon atom. Although more than 300 different AAs are found in nature, only 20 AAs are generally found as constituents of mammalian proteins^16^. In proteins, the amino and carboxyl groups are not available for chemical reactions as they are combined in peptide linkage. Thus, the role of an AA in a protein is governed by the nature of its side chain. AAs are categorized based on their R-group properties into nonpolar, polar, acidic, or basic AAs. From a nutritional perspective, AAs are classified as essential AAs (EAAs, obtained externally) and non-essential AAs (NEAAs, synthesized within the body). Metabolically, AAs can be converted into fat for energy storage (ketogenic AAs), oxidized into carbon dioxide and water for energy (glucogenic AAs), or exhibit both ketogenic and glucogenic properties^16^. While the Warburg effect has been recognized as a key contributor to endothelial-related diseases such as tumor angiogenesis^14,17^ and diabetic kidney disease^18^, its impact on AA metabolism in retinal endothelial cells under conditions of hyperglycemia and Hyp remains to be elucidated.

The objective of this study was to examine the AA metabolism in human retinal endothelial cells (HRECs) experiencing the Warburg effect induced by the combined influence of HG and Hyp. Additionally, the study aimed to identify the pathways enriched in dysregulated AAs and validate these findings in patients with PDR. The identification of these dysregulated AAs holds potential for druggable targets or biomarkers in PDR.

## Results

### Overall AA characteristics and their nutritional/metabolic profiles in HRECs cultured under normal conditions

**Figure 1:**
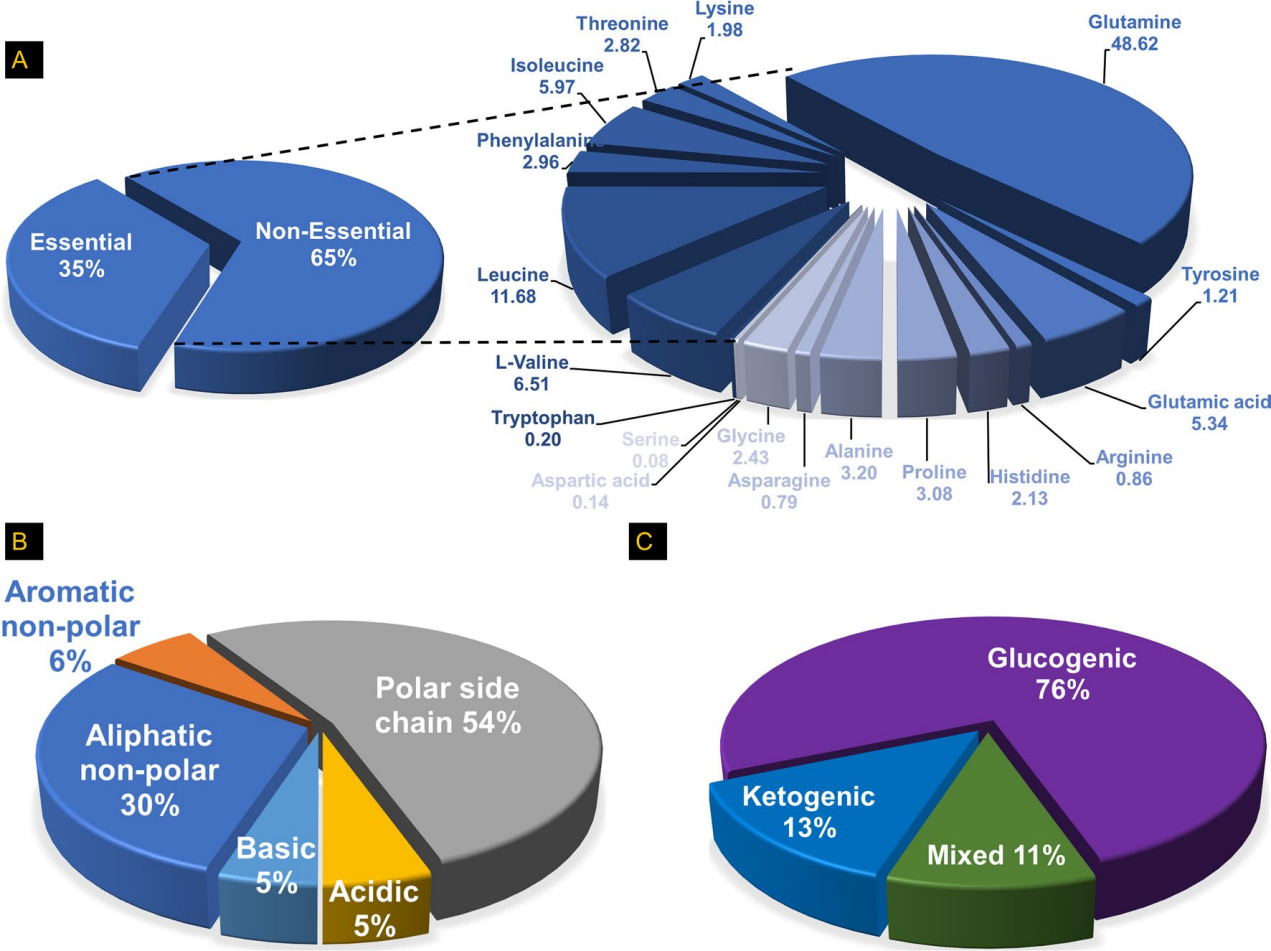
Profiles of amino acids (AAs) in human retinal endothelial cells (HRECs) cultured under normal conditions: The profiles are categorized as follows: (**A**) Nutritional classification: Non-essential AAs (glutamine, glutamic acid, alanine, proline, glycine, histidine, tyrosine, arginine, asparagine, aspartic acid, and serine) and essential AAs (leucine, valine, isoleucine, phenylalanine, threonine, lysine, and tryptophan); (**B**) Chemical classification: Aromatic/heterocyclic non-polar AAs (phenylalanine, proline, and tryptophan), polar (asparagine, glutamine, serine, and threonine, tyrosine), acidic (aspartic acid and glutamic), basic (arginine, lysine, and histidine), and aliphatic non-polar AAs (glycine, alanine, isoleucine, leucine, and valine); (**C**) Metabolic classification: Glucogenic AAs (glycine, alanine, serine, aspartic acid, glutamic acid, glutamine, valine, threonine, proline, histidine, asparagine, and arginine), ketogenic AAs (leucine and lysine), and mixed glucogenic and ketogenic AAs (phenylalanine, isoleucine, tryptophan, tyrosine).

We first characterized the profile of AAs in HRECs isolated from healthy donors and cultured under normal conditions using LC–MS/MS. Nutritionally, most AAs identified in HRECs were found to be non-essential (65%, Fig. 1A), and the most abundant non-essential AAs (from highest to lowest) were glutamine, glutamic acid, alanine, proline, glycine, histidine, tyrosine, arginine, asparagine, aspartic acid, and serine (Fig. 1A and supplement Table 1). On the other hand, essential AAs constitute 35% of the total AAs in HRECs (Fig. 1A), and the most abundant essential AAs (from highest to lowest) were leucine, valine, isoleucine, phenylalanine, threonine, lysine, and tryptophan (supplement Table 1). From a structural perspective, the majority of AAs in HRECs had polar side chains (54%), followed by aliphatic non-polar side chains (30%). A smaller proportion consisted of aromatic/heterocycle non-polar side chains (6%), while basic and acidic side chains each constituted 5% (Fig. 1B). Regarding metabolism, ketogenic AAs accounted for 13% of the identified amino acids, while mixed ketogenic or glucogenic AAs represented 11% (Fig. 1C). Importantly, a significant proportion of the identified AAs in HRECs were found to be glucogenic, making up 76% of the total AAs (Fig. 1C), suggesting a potential association between the Warburg effect and AAs metabolism in HRECs.

### Amino acid profiles of HRECs cultured under the Warburg effect induced by the combined effects of high glucose (HG) and hypoxia (Hyp)

**Figure 2:**
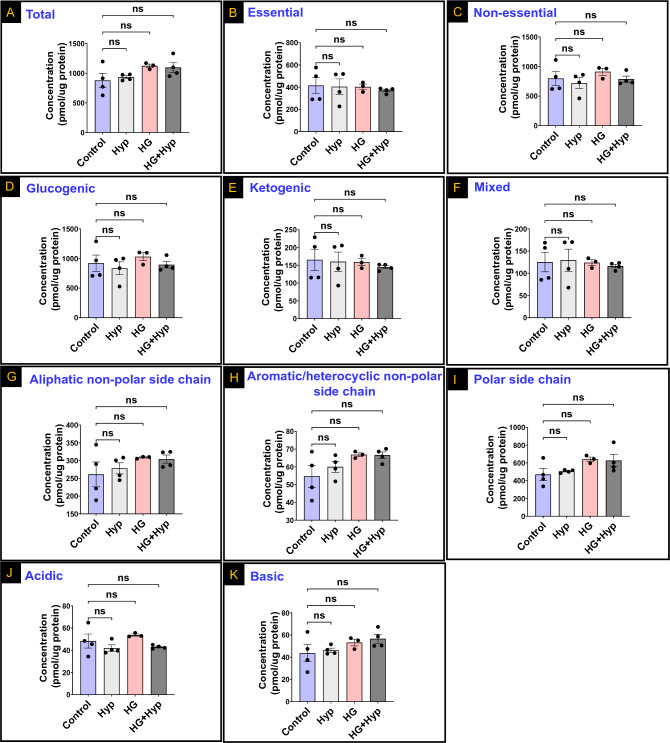
Levels of (**A**) Total amino acids (AAs); (**B**) Essential AAs; (**C**) Non-essential AAs; (**D**) Glucogenic AAs; (**E**) Ketogenic AAs; (**F**) Mixed ketogenic and glucogenic AAs; (**G**) Aliphatic non-polar side chain AAs; (**H**) Aromatic/heterocyclic non-polar side chain AAs; (**I**) Polar side chain AAs; (**J**) Acidic side chain AAs; and (**K**) Basic side chain AAs in human retinal endothelial cells (HRECs) treated with osmotic control (Mannitol; 25 mM) or high glucose (HG; 25 mM) for 5 days; followed by normoxia or hypoxia (Hyp; 2% O_2_ and 5% CO_2_) for 24 h. Data are mean ± SD; n = 3–4/group; and ns: no significance. Comparisons between groups were made by ANOVA followed by Sidak's post-test.

To characterize the AA profile in HRECs cultured under HG and Hyp, we first conducted experiments to confirm the induction of the Warburg effect under these conditions. Lactate production as well as glucose uptake were measured as markers of the induction of the Warburg effect in response to HG, Hyp, or their combination. The results obtained in Fig. S1A and B showed a significant increase in lactate production and glucose uptake compared to the control group, confirming the induction of the Warburg effect. Importantly, the induction of the Warburg effect did not lead to cell death, as evidenced by the evaluation of lactate dehydrogenase (LDH) release from HRECs (Fig. S2). Next, we used LC–MS/MS to investigate the total concentrations of all AAs in the different treatment groups, including HG alone, Hyp alone, HG + Hyp, and normal conditions. Surprisingly, we did not observe any significant global differences in the concentrations of total AAs among these groups (Fig. 2A). Our observations remained consistent when examining the stratification of AAs based on nutritional value into essential and non-essential AAs as no significant differences observed between the HG + Hyp group and the Hyp, HG, or control groups (Fig. 2B and C, respectively). Similarly, when AAs were categorized broadly according to their metabolic fate as glucogenic, ketogenic, or mixed glucogenic and ketogenic, no significant differences were found between the HG + Hyp group and the Hyp, HG, or control groups (Fig. 2D, E, and F, respectively). Furthermore, when AAs were grouped based on their side chain characteristics, including aliphatic non-polar, aromatic/heterocyclic non-polar, polar, acidic, or basic side chains, no significant differences were observed between the HG + Hyp group and the Hyp, HG, or control groups (Fig. 2G, H, I, J, and K, respectively). However, it is worth noting that there was an increasing trend in the levels of AAs with non-polar, polar, and basic side chains in the HG + Hyp group compared to the control group, which led us to investigate further such differences as follows.

### Chemical profiles of AAs adjusted for the nutritional value in HRECs cultured under the Warburg effect induced by the combination of HG and Hyp

**Figure 3:**
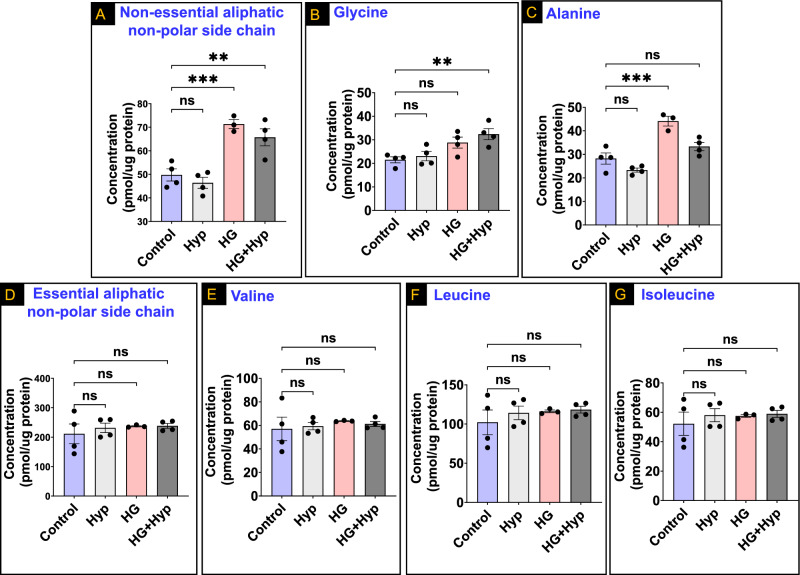
Levels of (**A**) Total non-essential amino acids (AAs) with aliphatic non-polar side chains, (**B**) Glycine, (**C**) Alanine, (**D**) Total essential AAs with aliphatic non-polar side chains, (**E**) Valine, (**F**) Leucine, and (**G**) Isoleucine in human retinal endothelial cells (HRECs) treated with osmotic control (Mannitol; 25 mM) or high glucose (HG; 25 mM) for 5 days; followed by normoxia or hypoxia (Hyp; 2% O_2_ and 5% CO_2_) for 24 h. Data are mean ± SD; n = 3–4/group; ns: no significance; **: *P* < 0.01; and ***: *P* < 0.001. Comparisons between groups were made by ANOVA followed by Sidak's post-test.

To further investigate potential variations in AA profiles between HRECs cultured under the Warburg effect induced by HG and Hyp compared to other HREC groups, the chemical profiles of AAs were adjusted to account for their nutritional significance. To achieve this, each subgroup of amino acids within the chemical classification was further categorized into essential and non-essential amino acids within their respective subgroups. The analysis revealed notable differences in the concentrations of AAs with aliphatic non-polar side chains under the influence of the Warburg effect after adjusting for nutritional value. Specifically, significantly higher levels of non-essential AAs with aliphatic non-polar side chains were observed in the HG + Hyp group compared to the control group as well as in the HG group compared to the control group. However, no such differences were found between the Hyp and control groups (Fig. 3A). Further analysis showed specific AA enrichments within the HG + Hyp and HG groups. Glycine exhibited a significant increase in concentration in the HG + Hyp group compared to the control group (Fig. 3B), while alanine levels were significantly elevated in the HG group compared to the control group (Fig. 3C). Glycine levels remained similar between the Hyp and control groups, as well as between the HG and control groups. Similarly, alanine levels showed no significant differences between the Hyp and control groups, as well as between the HG + Hyp and control groups. In contrast to non-essential AAs in the category of AAs with aliphatic non-polar side chains, there were no significant differences observed in the total concentrations of essential AAs within this category among all experimental groups (Fig. 3D). This lack of association remained consistent even when examining the individual AAs: valine, leucine, and isoleucine (Fig. 3E, F, and G, respectively). Taken together, these results indicate a differential impact of the Warburg effect induced by HG + Hyp compared to the Warburg effect induced by HG or Hyp alone on the metabolism of AAs with aliphatic non-polar side chains, particularly when considering their nutritional value.

**Figure 4:**
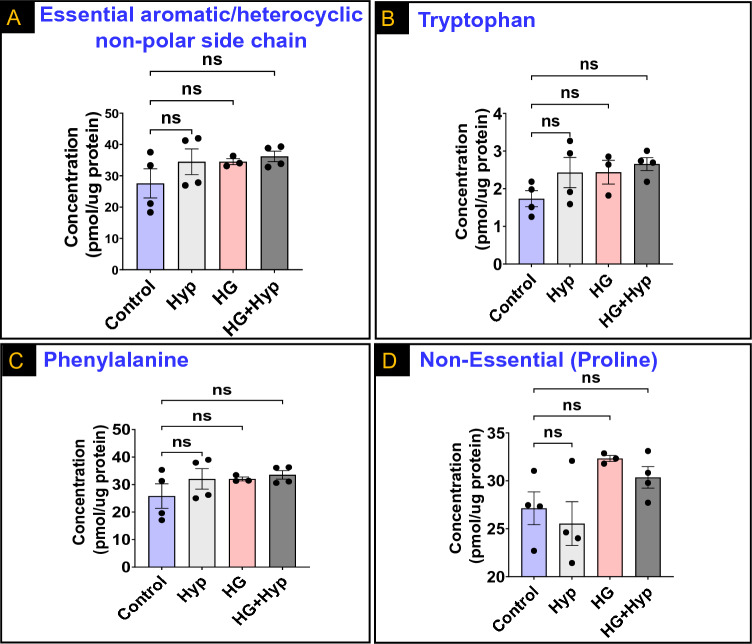
Levels of (**A**) Total essential amino acids (AAs) with aromatic/heterocycle non-polar side chains; (**B**) Tryptophan; (**C**) Phenylalanine; and (**D**) Non-essential AA with a heterocycle non-polar side chain (Proline) in human retinal endothelial cells (HRECs) treated with osmotic control (Mannitol; 25 mM) or high glucose (HG; 25 mM) for 5 days; followed by normoxia or hypoxia (Hyp; 2% O_2_ and 5% CO_2_) for 24 h. Data are mean ± SD; n = 3–4/group; and ns: no significance. Comparisons between groups were made by ANOVA followed by Sidak's post-test.

Next, we adjusted AAs with aromatic/heterocyclic non-polar side chains for nutritional value. We found that the levels of essential AAs in this category were similar between HRECs experiencing the Warburg effect in the HG + Hyp group and the other experimental groups (Fig. 4A). This similarity also extended to individual AAs within the group, including tryptophan and phenylalanine (Fig. 4B and C, respectively). Additionally, proline, a non-essential AA within this category that contains an α-imino group, exhibited comparable concentrations in HRECs subjected to HG + Hyp compared to HRECs in the control group (Fig. 4D). These findings suggest that the metabolic alterations associated with the Warburg effect do not significantly impact the levels of AAs with aromatic/heterocyclic non-polar side chains, even after adjusting for nutritional value.

**Figure 5:**
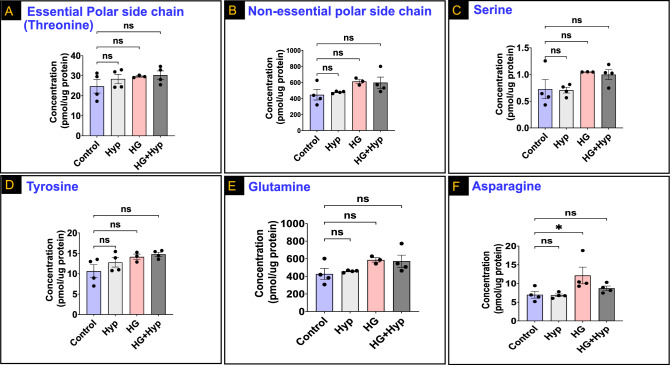
Levels of (**A**) Essential amino acids (AAs) with polar side chain (threonine); (**B**) Non-essential polar side chain AAs; (**C**) Serine; (**D**) Tyrosine; (**E**) Glutamine; and (**F**) Asparagine in human retinal endothelial cells (HRECs) treated with osmotic control (Mannitol; 25 mM) or high glucose (HG; 25 mM) for 5 days; followed by normoxia or hypoxia (Hyp; 2% O_2_ and 5% CO_2_) for 24 h. Data are mean ± SD; n = 3–4/group; ns: no significance; and *: *P* < 0.05. Comparisons between groups were made by ANOVA followed by Sidak's post-test.

In a similar manner, after adjusting for nutritional value, no significant differences were observed in AAs with polar side chains under the influence of the Warburg effect across all experimental groups [Fig. 5A and B for essential (threonine only in this group) and non-essential AAs, respectively]. This lack of association remained consistent even when examining individual non-essential AAs within this group, including serine, tyrosine, and glutamine (Fig. 5C, D, and E, respectively). However, there was a significant increase in the levels of asparagine in the HG group compared to the control group, while its concentrations remained similar in the HG + Hyp, Hyp, and control groups (Fig. 5F). These findings suggest that the Warburg effect does not significantly affect the levels of AAs with polar side chains, with the exception of asparagine, which showed an increase specifically in the HG group.

**Figure 6:**
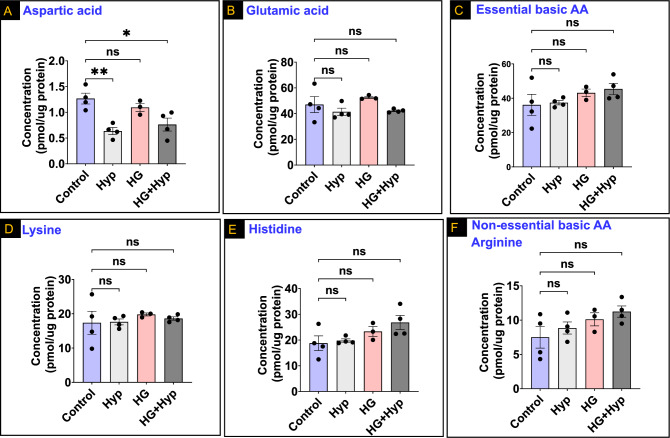
Levels of (**A**) Aspartic acid; (**B**) Glutamic acid; (**C**) Essential basic amino acids (AAs); (**D**) Lysine; (**E**) Histidine; and (**F**) Non-essential basic AA (arginine) in human retinal endothelial cells (HRECs) treated with osmotic control (Mannitol; 25 mM) or high glucose (HG; 25 mM) for 5 days; followed by normoxia or hypoxia (Hyp; 2% O_2_ and 5% CO_2_) for 24 h. Data are mean ± SD; n = 3–4/group; ns: no significance; *: *P* < 0.05; and **: *P* < 0.01. Comparisons between groups were made by ANOVA followed by Sidak's post-test.

Next, we investigated the impact of the Warburg effect on AAs with acidic side chains in HRECs. Intriguingly, aspartic acid exhibited a decrease in response to Hyp, with significantly lower levels observed in both the Hyp and HG + Hyp conditions compared to the control group (Fig. 6A). In contrast, glutamic acid, which shares structural similarities with aspartic acid, did not show any significant differences in its concentrations among the three treatment groups of HRECs compared to the control group (Fig. 6B). These findings suggest a specific effect of Hyp on the levels of aspartic acid, indicating its potential involvement in the cellular response to hypoxic conditions, while glutamic acid appears to be less affected in this context.

Finally, AAs with basic side chains showed consistent concentrations under the Warburg effect across all experimental groups, even after considering their nutritional value (Fig. 6C). For example, essential AAs within this category, such as lysine and histidine, displayed similar levels without significant differences among the experimental groups (Fig. 6D and E, respectively). Likewise, arginine, a non-essential AA with a basic side chain, exhibited comparable concentrations among the experimental groups of HRECs experiencing the Warburg effect when compared to the control group (Fig. 6F). These findings suggest that the Warburg effect does not significantly impact the concentrations of AAs with basic side chains, regardless of their essential or non-essential status.

Taken together, these findings suggest that there is no global alteration in AA metabolism in HRECs under the Warburg effect induced by HG and/or Hyp conditions. However, when the chemical profile of AAs adjusted for nutritional value, specific individual AA metabolism appears to be differentially influenced by the Warburg effect associated with each inducer.

### Pathway analysis of AAs in HRECs cultured under the Warburg effect induced by the combination of HG and Hyp

**Figure 7:**
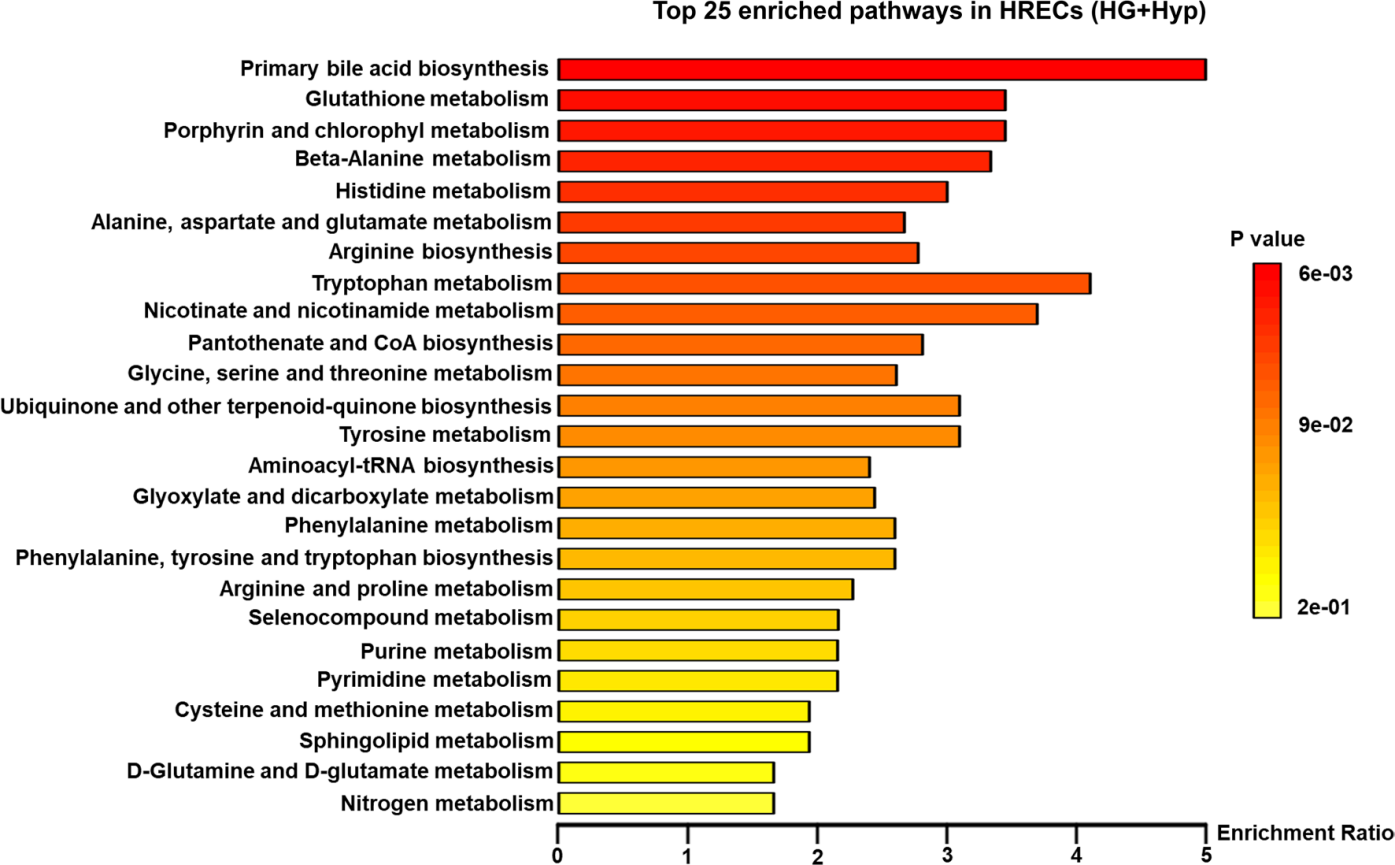
Top 25 enriched KEGG pathways in human retinal endothelial cells (HRECs) subjected to HG + Hyp vs. mannitol control. KEGG: Kyoto Encyclopedia of Genes and Genomes. HG + Hyp: high glucose with hypoxia-treated HREC group.

Pathway analysis of AAs was performed to identify relevant metabolic pathways affected by the Warburg effect induced by HG and Hyp. The analysis revealed that 25 KEGG pathways exhibited significant enrichment (*p* value < 0.05) under the influence of the Warburg effect between the HG + Hyp and control groups, including the pathways involved in cell proliferation, such as nucleotide biosynthesis and nitrogen metabolism (Fig. 7). Notably, glycine emerged as a crucial component within these pathways, playing a key role in synthesizing intermediate products associated with these metabolic pathways, particularly those linked to angiogenesis and cell proliferation, such as purine and pyrimidine biosynthesis.

### Glycine levels in the vitreous humor derived from patients with PDR

**Figure 8:**
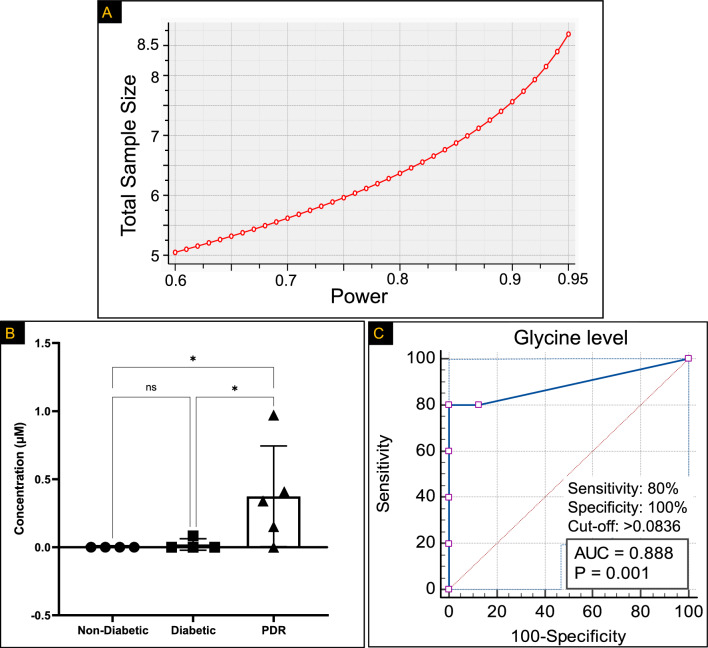
(**A**) The sample size was calculated based on the power analysis of glycine data from Fig. 3B. A sample size of 4–5 patients per group provides at least 90–95% power in delineating the anticipated differences. (**B**) The analysis of glycine in vitreous samples using LC–MS/MS demonstrates that the proliferative diabetic retinopathy (PDR) group exhibits the highest occurrence of glycine compared to both control groups. These control groups include patients with diabetes but without PDR, as well as patients without diabetes or PDR. The data within the control group did not pass the Shapiro–Wilk test for normality. Consequently, group comparisons were conducted using the non-parametric Kruskal–Wallis test. (**C**) Receiver operating characteristic (ROC) curve of optimal cut-off value characteristics for dichotomizing patients into high or low glycine levels. This curve assesses the diagnostic performance of glycine as a predictor. Data are mean ± SD; n = 4–5/group.

For translational purpose, we next aimed to investigate the association between glycine and PDR. To achieve this goal, we have first measured the levels of glycine in the vitreous samples derived from PDR patients versus control groups including patients with diabetes but no clinical evidence of PDR and patients without diabetes, all of whom underwent pars plana vitrectomy (PPV) for epiretinal membrane or macular hole. The sample size was calculated based on the power analysis of glycine data from Fig. 3B, where glycine levels were significantly increased in HRECs exposed to the Warburg effect induced by the dual effects of HG and Hyp [32.4 ± 4.7 vs. 21.5 ± 2.5 (control), pmol.µg protein, mean ± SD]. The power analysis was conducted using G*Power 3.1.9.4 software utilizing a priori type of power analysis in which the sample size (n) was computed as a function of the desired power level (1-β), a significance level (α) of 0.05 (two-tail), and the expected effect size (d) calculated from the mean and standard deviation of each group. As depicted in Fig. 8A, a sample size of 4–5 individuals per group provided at least 90–95% power to detect the anticipated differences. Using this sample size, LC–MS/MS analysis of the vitreous samples revealed that the PDR group had the highest incidence of glycine. This distinction was statistically significant when compared to both individuals with diabetes but without PDR (0.3740 µM ± 0.37 SD vs. 0.021 µM ± 0.041 SD, respectively; *p* < 0.05), and individuals without diabetes or PDR, where glycine levels in the vitreous humor samples were undetectable (Fig. 8B).

Secondly, after having shown that glycine level was increased significantly among PDR patients, we next sought to quantify the strength of the association between presence of glycine and PDR. Therefore, odds ratios were calculated by comparing the odds of having a high glycine level in PDR cases to the odds in non-PDR controls. In this regard, the samples were dichotomized into high or low glycine levels based on a predetermined cutoff value. The cutoff limit was determined using receiver operating characteristic (ROC) curves, analyzing the levels and distribution of glycine in control and PDR vitreous samples. The ROC analysis yielded an area under the curve (AUC) of 0.888 (SE = 0.133, *P* < 0.0006, 95% CI: 0.595–0.992). The optimal cutoff criterion maximizing sensitivity and specificity for detecting glycine in PDR patients was determined to be 0.0836 µM, based on the large number of detection criteria (Fig. 8C). At this concentration, the sensitivity was 80% and specificity was 100%. Based on this classification, the odds ratio for an association between the presence of glycine and PDR was calculated. The odds ratio for PDR patients presenting glycine levels above the cutoff of 0.0836 µM was 28 (with a p value of 0.0312) compared to non-PDR controls (supplement Table 2), suggesting strong association between PDR and high glycine levels.

## Discussion

The main finding of this study is that there is no global alteration in AA metabolism in HRECs due to the Warburg effect induced by the risk factors associated with PDR, namely HG and Hyp conditions. However, when considering the adjusted chemical profile of AAs based on their nutritional value, specific individual AA metabolism appears to be differentially impacted by the Warburg effect associated with each inducer. Particularly, the homeostasis of non-essential AAs with aliphatic non-polar side chains, specifically glycine, is dysregulated under the influence of the combination of HG and Hyp conditions, common risk factors known to induce the Warburg effect and to be involved in the PDR pathogenesis. In support, significantly higher levels of glycine were found in vitreous samples obtained from patients with PDR compared to those from non-diabetic and diabetics without PDR. This is further corroborated by the presence of a strong association between PDR and high glycine levels, as indicated by a high odds ratio showing that PDR is more frequent in patients presenting with high glycine levels.

It has been shown that glycine exerts its biochemical effects via specific glycine receptors (GlyRs), which are present in various cell types, including endothelial cells^19,20^. However, there is contradictory evidence regarding the role of glycine in regulating endothelial biology and function. For example, Yu et al. examined the relationship between glycine and endothelial functionality and found that glycine promoted endothelial cell proliferation and survival, but repressed angiogenesis^21^. Conversely, Guo et al. showed that VEGF signaling requires glycine to induce endothelial angiogenesis via the glycine transporter (GlyT)1/glycine-mammalian target of rapamycin (mTOR)/voltage-dependent anion channel (VDAC)-1 axis pathway^22^. These contradicting data can be reconciled by considering the concentration of glycine, which showed a dose-dependent biphasic effect on angiogenesis: low glycine concentration (below 100 mM) promotes new blood vessel formation, while high glycine concentration (400 mM and above) induces anti-angiogenesis^23^. In our study, we found the glycine concentration in vitreous samples obtained from individuals with PDR falls within the range that promotes angiogenesis. The mean detected glycine level we measured was 0.3740 µM ± 0.37 SD, accompanied by a 25% interquartile range of 0.075 µM and a 75% interquartile range of 0.68 µM. The confidence intervals were determined to span from -0.08 µM to 0.8334 µM. It is noteworthy that none of the vitreous samples acquired from non-diabetic control patients lacking PDR displayed any detectable glycine level. Nonetheless, the significance of these findings in terms of assessing the effects of glycine on HRECs' proliferation, functionality, and metabolic behavior needs to be further investigated.

In PDR patients, retinal homeostasis is reflected in changes in vitreous biochemistry^24^; however, few studies reported the role of glycine in PDR. Vidhya et al.^25^ reported that compared with control samples, glycine was increased in PDR vitreous samples (N = 30). Likewise, Tomita et al.^26^ found that glycine was elevated in the vitreous of PDR patients (N = 43) compared with controls. Consistent with these findings, our study also showed that glycine was increased in PDR vitreous humor compared with control participants. However, our study is the first to investigate the link between glycine levels and PDR using a ROC-based approach. This approach allowed us to determine the operational detection limit of glycine in PDR and establish its utility as a biomarker for the disease. Our results indicated that detecting glycine above 0.08 µM in vitreous samples yielded high sensitivity (80%) and specificity (100%) for diagnosing PDR. Furthermore, our research provided novel insights into the underlying mechanisms contributing to elevated glycine levels. Through a comparison of the impact of the Warburg effect induced by HG alone, Hyp alone, and the combined effects of Hyp and HG on AA metabolism in HRECs, we observed distinct subtypes despite the shared outcome of increased lactate production—an observation previously reported in the vitreous of PDR patients^26–28^. Our study demonstrated that the specific subtype of the Warburg effect induced by the combination of Hyp and HG plays a critical role in redirecting glycolytic intermediates towards the accumulation of glycine. This accumulation of glycine is involved in the synthesis of purine and pyrimidine, as indicated by pathway analysis conducted in HRECs (Fig. 7). These processes are pivotal steps in generating the nucleotides required for nucleic acid synthesis during cellular proliferation and angiogenesis^29,30^.

It is worth mentioning that in the presence of hyperglycemic conditions, there is an augmented flow of glucose through the glycolytic pathway, causing cells to undergo hyperglycolysis. This state is characterized by a substantial glycolytic flux directed towards the production of pyruvate^31^. Subsequently, pyruvate can undergo various transformations^32^, including conversion into lactate by lactate dehydrogenase, conversion into alanine through the action of alanine transaminase (ALT), or transportation into the mitochondria via the mitochondrial pyruvate carrier (MPC)^33^. Once inside the mitochondria, pyruvate enters the Krebs cycle, ultimately resulting in the generation of aspartic acid^34^. Furthermore, aspartic acid can be further converted into asparagine through the activity of asparagine synthase^35^. In support of this concept, our observations demonstrate significant increases in the alanine, asparagine, and lactate levels (Figs. 3C, 5F, and S1, respectively) when inducing a specific subtype of the Warburg effect in HRECs using HG alone. On the other hand, when mitochondrial function is impeded by hypoxia, pyruvate is unable to enter the Krebs cycle and generate aspartic acid, resulting in decreased aspartic acid levels. Instead, pyruvate is forced to convert into lactate^36^. In accordance with this, Fig. 6A illustrates that hypoxia reduces aspartic acid levels in HRECs while increasing lactate production (Fig. S1), thus creating a distinct subtype of the Warburg effect. It is important to note that blocking mitochondrial activity through hypoxia under hyperglycemic conditions induces an imbalanced state of glycolysis. In this state, upper glycolysis (where ATP is invested) outpaces lower glycolysis (where ATP is produced), leading to the accumulation of the glycolytic intermediate 3-phosphoglycerate (G3P)^37^. Since G3P serves as a substrate for both lower glycolysis and glycine biosynthesis, its increase tends to enhance lactate production (as shown in Fig. S1), while simultaneously promoting its conversion into glycine (as shown in Fig. 3B). This phenomenon gives rise to a specific subtype of the Warburg effect, referred to as the Warburg effect induced by HG + Hyp.

One limitation of our study is the absence of a comparison between the AA profiles in the serum or aqueous humor and the vitreous humor within the same patient. Future studies should include a larger cohort of patients with diabetes as well as PDR and compare the levels of glycine across vitreous and aqueous humors as well as in the blood. The significance of this approach lies in the accessibility and reduced invasiveness of both aqueous humor and blood samples. Evaluating potential correlations of glycine levels among these three sample types will play a crucial role in shaping the development of clinical screening tools aimed at enhancing the detection of PDR.

Overall, our study not only strengthens the evidence of elevated glycine levels in PDR but also unveils a novel mechanistic understanding of how a specific subtype of the Warburg effect contributes to glycine accumulation. These findings contribute to the potential use of glycine as a biomarker and deepen our comprehension of PDR pathogenesis.

## Materials and methods

### Cell culture

Human retinal endothelial cells (HRECs) were procured from Cell Systems (Kirkland, WA, USA) and cultured, as previously described^38,39^, in Microvascular Endothelial Cell Growth Medium-2 Bullet Kit (Lonza, Walkersville, MD, USA; Catalog No. CC-3202 EGM-2 MV), which includes base medium and growth kit supplements, containing human fibroblast growth factor (hFGF), human vascular endothelial growth factor (hVEGF), human insulin-like growth factor (hIGF-1), human epidermal growth factor (hEGF), and 5% fetal bovine serum (FBS). HRECs of passage 3–6 were seeded in 100 mm Petri dishes until they reached 90% confluency, then the medium was changed to fresh medium containing 5% FBS, but without growth factors, that had either mannitol (25 mM), which was the osmotic control group, or HG (25 mM) for 5 days. Hypoxia (Hyp) was introduced 24 h before cell harvesting by incubating HRECs in a 2% O_2_ humidified hypoxic chamber with concurrent 5% CO_2_.

### Harvesting HRECs for LC–MS/MS-based targeted metabolomics

For harvesting HRECs, the culture media were removed and the cells were rinsed with warm PBS. The petri dishes containing HRECs were placed on top of liquid nitrogen to quench the metabolism of the cells immediately. Then, 1 mL of 80% pre-chilled MeOH was added into each petri dish to further quench the metabolism of HRECs and to extract intracellular metabolites. HRECs were scraped and transferred to 1.5 mL centrifuge tubes. The lysed cells were stored at − 80 °C until metabolomic analyses were performed.

### Patients and clinical samples collection

This study adhered to the principles outlined in the Declaration of Helsinki and received approval from the Institutional Review Board of Wayne State University (IRB#: 090319MP2E). Vitreous samples, ranging from 0.5 to 1 ml, were collected from patients diagnosed with PDR (mean age: 59.1 ± 14.0 years). Control groups included patients with diabetes but no clinical evidence of PDR (mean age: 74.0 ± 10.9 years) and patients without diabetes (mean age: 66.2 ± 2.3 years), all of whom underwent PPV for conditions such as epiretinal membrane or macular hole. Written informed consent for the collection of the vitreous samples during PPV was obtained from all patients. The clinical characteristics of the patients from which the vitreous humor was obtained are described in supplement Table 3. Vitreous samples were immediately frozen in liquid nitrogen and then stored at − 80 °C. Thereafter, 1 mL of 80% pre-chilled MeOH was added to 100 µL of the vitreous sample to extract the metabolites, which were then subjected to the AA analysis as described below.

### Liquid chromatography-tandem mass spectrometry (LC–MS)/MS-based targeted analysis for AAs

A targeted metabolomics approach using LC–MS/MS was employed to investigate the intracellular AA metabolism in HRECs experiencing the Warburg effect induced by HG and Hyp. The analysis was performed on an AB SCIEX QTRAP 6500 LC–MS/MS system, consisting of a SHIMADZU Nexera ultra-high-performance liquid chromatography (UHPLC) coupled with a triple quadrupole/linear ion trap mass spectrometer. The LC-/MS system control and data acquisition were managed using analyst 1.6 software, while data processing and quantitation utilized Multiquanta 3.0 software. AAs present in HRECs as well as in human vitreous samples were extracted based on the method described previously by Bao^40^ with minor modifications. Briefly, 1 mL of prechilled MeOH (cooled at − 80 °C) was first added to the harvested HRECs followed by vortex-mixing and centrifugation at 10,000 rpm at 4 °C for 10 min. The resulting supernatant was collected and transferred into a new 2 mL microcentrifuge tube. Then, 0.8 mL of prechilled 80% MeOH was added followed by vortex-mixing and centrifugation in the same condition performed in the earlier step. Following this step, the combined supernatants of each sample were dried in a CentriVap Refrigerated Centrifugal Concentrator (Kansas City, Missouri) at 10 °C. For chromatographic separation, the residual was first reconstituted in a mixture containing 125 μL of acetonitrile: water (50:50, v/v). Then, the samples were properly mixed by vortex before centrifugation. The resulting supernatant of each sample was further diluted, and then subjected to LC–MS/MS analyses. Two runs of LC–MS/MS were performed based on different chromatographic separation mechanisms, reverse phase liquid chromatography (separates nonpolar amino acids based on their hydrophobicity) and hydrophilic-interaction liquid chromatography (separates highly polar amino acids). Reversed phase liquid chromatography (RPLC) was performed using Synergi Polar-RP column (80 A, 2.0 × 150 mm, 4 μm). The mobile phases for chromatographic separation consisted of the mobile phase A (0.03% formic acid in water) and the mobile phase B (0.03% formic acid in acetonitrile). The flow rate was set constant at 0.25 mL/min. The gradient program was set as follows: 0–0.3 (0% B), 0.3–25 min (0–95% B), 25–25.1 min (95–0% B) and 25.1–30 min (0% B), while the hydrophilic-interaction liquid chromatography (HILIC) was performed using Atlantis HILIC Silica column (2.1 × 150 mm, 3 μm). The mobile phases for chromatographic separation consisted of the mobile phase A (10 mM ammonium formate, pH 3.0, and 0.1% formic acid in water) and the mobile phase B (0.1% formic acid in acetonitrile). The flow rate was set constant at 0.25 mL/min. The gradient was programmed as follows: 0–0.5 min (95% B), 0.5–10.5 min (95–40% B), 10.5–15 min (40% B), 15–17 min (40–95% B) 17–24 min (95% B). The column eluents were monitored using multi-reaction monitoring (MRM) on a QTRAP 6500 mass spectrometer under positive and negative ionization mode. MS parameters, viz. ionization polarity, product ion, collision energy, declustering potential, and cell exit potential, were optimized to obtain the most sensitive and specific mass transitions for individual metabolites, and the standard solutions used for calibration. Furthermore, certain MS parameters were also optimized: ion spray potential at 5500 V for positive ionization mode and 4500 V for negative ionization mode; nebulizer gas and bath gas at 50 psi; curtain gas at 30 psi; collision gas at medium level; and source temperature at 475 °C. The dwell time for each MRM transition was 3 ms for the both the reversed-phase and HILIC methods. For every individual AA, a corresponding calibration standard (10 nM to 10 μM) was prepared in the appropriate mobile phase. For quality control, control samples (200 nM) were prepared in the suitable mobile phase. As the concentration of intracellular amino acids in cells may vary, both original samples and 20-fold diluted samples were processed and subjected to LC–MS/MS analyses. The normalization of AA levels in HRECs was achieved by considering the protein concentration of each sample.

### Data analysis

Statistical tests for the comparisons of the levels of AAs between groups were two-sided, with significance set at *p* < 0.05. Data were examined for normality distribution using the Shapiro–Wilk test. If the data passed the Shapiro–Wilk test for normality, comparisons between groups were made by ANOVA followed by Sidak's post-test (multiple groups). If data failed the Shapiro–Wilk test, the non-parametric Kruskal–Wallis test was used. In addition, AAs were analyzed by the MetaboAnalyst, a web-based statistical package (http://www.metaboanalyst.ca/), where the pathway enrichment analysis was performed using the Kyoto Encyclopedia of Genes and Genomes (KEGG) pathway enrichment tool^41–43^. Receiver operating characteristic (ROC) curve analysis and odds ratios were conducted using MedCalc statistical software for biomedical research.

### Supplementary Information

Supplementary Information.

## Data Availability

Data associated with this manuscript are available upon request from corresponding author.
