# Supplementary material for: The Warburg effect alters amino acid homeostasis in human retinal endothelial cells: implication for proliferative diabetic retinopathy
Source: Sci Rep. 2023 Sep 25;13:15973. doi: 10.1038/s41598-023-43022-z (PMC10520048; doi:10.1038/s41598-023-43022-z)
Supplement: Supplementary file 1 — Supplementary Information. [file 41598_2023_43022_MOESM1_ESM.docx]

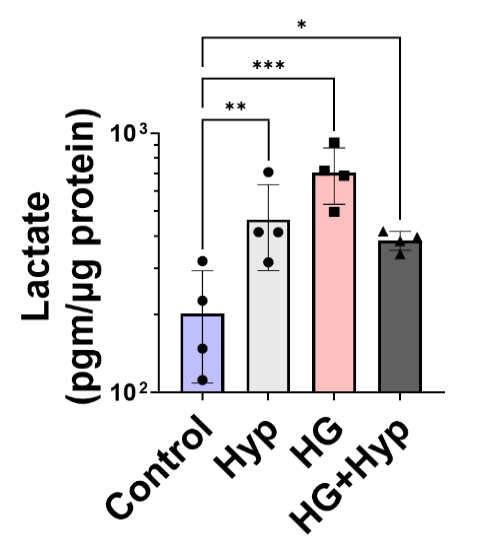


B

A

**Figure S1. The induction of the Warburg effect in human retinal endothelial cells (HRECs), as assessed by lactate production (A) and intracellular glucose accumulation (B).** The experiment involved subjecting HRECs to either an osmotic control (Mannitol; 25 mM) or high glucose (HG; 25 mM) treatment for a duration of 5 days. Subsequently, the cells were exposed to either normoxia or hypoxia (Hyp; 2% O_2_ with 5% CO_2_) for a period of 24 hours. The presented data represents the mean±SD of four replicates per group. Significance levels are denoted as follows: * for P<0.05, ** for P<0.01, and *** for P<0.001.

**Figure S2. The induction of the Warburg effect in human retinal endothelial cells (HRECs) did not cause cell death as assessed by the release of lactate dehydrogenase (LDH) from the cells.** HRECs were treated with Mannitol (25 mM; osmotic control) or high glucose (HG; 25 mM) for a duration of 5 days. Then, the cells were exposed to either normoxia or hypoxia (Hyp; 2% O_2_ with 5% CO_2_) for a period of 24 hours. The presented data represents the mean±SD of four replicates per group. Nor: Normalized; ns: not significant.

**Table 1.** The abundance of amino acids in HRECs.

| **Non-Essential Amino Acid** | **%** | **Essential Amino Acid** | **%** |
| --- | --- | --- | --- |
| Glutamine | 48.6 | Leucine | 11.7 |
| Glutamic acid | 5.3 | Valine | 6.5 |
| Alanine | 3.2 | Isoleucine | 6.0 |
| Proline | 3.1 | phenylalanine | 3.0 |
| Glycine | 2.4 | Threonine | 2.8 |
| Histidine | 2.1 | Lysine | 2.0 |
| Tyrosine | 1.2 | Tryptophan | 0.2 |
| Arginine | 0.9 |  |  |
| Asparagine | 0.8 |  |  |
| Aspartic acid | 0.1 |  |  |
| Serine | 0.1 |  |  |

**Table 2.** Rate of proliferative diabetic retinopathy (PDR) in patients presenting vitreous glycine levels above or below the cut-off of 0.0836 µM (calculated by the ROC curves) compared with controls (non-PDR) comprised of both patients with diabetes but without PDR, as well as patients without diabetes or PDR.

| **Glycine level** | **PDR** | | **Non-PDR** | | **OR^a^** |
| --- | --- | --- | --- | --- | --- |
|  | **n** | **%** | **n** | **%** |  |
| **High** | 4 | 80^b^ | 1 | 12.5 | 28 |
| **Low** | 1 | 20 | 7 | 87.5 |  |

a: OR relative to an increase of 1 standard deviation among controls.

b: p< 0.0312 compared to the non-PDR group.

OR: Odd ratio.

**Table 3.** Clinical characteristics of the patients from which vitreous humor was obtained from

| **Diabetes status** | **Age** | **Sex** | **Race** | **Eye** | **Type 1 or Type 2 DM** |
| --- | --- | --- | --- | --- | --- |
| Control (non-diabetic) | 65 | Female | AAm | OD | N/A |
|  | 68 | Male | AAm | OD | N/A |
|  | 67 | Male | Caucasian | OD | N/A |
|  | 63 | Male | AAm | OS | N/A |
| Diabetes without PDR | 58 | Female | NHW | OS | Type 2 |
|  | 84 | Male | Caucasian | OD | Type 2 |
|  | 76 | Male | AAm | OS | Type 2 |
|  | 76 | Male | Caucasian | OS | Type 2 |
| PDR | 64 | Male | AAm | OS | Type 2 |
|  | 56 | Male | AAm | OS | Type 2 |
|  | 62 | Male | AAm | OD | Type 2 |
|  | 74 | Female | Caucasian | OD | Type 2 |
|  | 37 | Male | AAm | OD | Type 1 |
| Abbreviations: AAm = African-American; DM = diabetes mellitus; N/A = not applicable; NHW = non-Hispanic White; OD = right eye; OS = left eye; PDR = proliferative diabetic retinopathy. | | | | | |
